# Supplementary material for: Magnetic field boosted ferroptosis-like cell death and responsive MRI using hybrid vesicles for cancer immunotherapy
Source: Nat Commun. 2020 Jul 20;11:3637. doi: 10.1038/s41467-020-17380-5 (PMC7371635; doi:10.1038/s41467-020-17380-5)
Supplement: Supplementary file 1 — Supplementary Information [file 41467_2020_17380_MOESM1_ESM.pdf]

Supplementary information

Magnetic Field Boosted Ferroptosis-like Cell Death and Responsive MRI Using Hybrid Vesicles for Cancer Immunotherapy

Yu et al.

Magnetic Field Boosted Ferroptosis-like Cell Death and Responsive MRI Using Hybrid Vesicles for Cancer Immunotherapy

Bo Yu[1], Bongseo Choi[1], Weiguo Li[1,2] and Dong-Hyun Kim[1,2,3,4]*

[1]Department of Radiology, Feinberg School of Medicine, Northwestern University, Chicago, IL 60611, USA

[2]Department of Bioengineering, University of Illinois at Chicago, Chicago, IL 60607, USA

[3]Robert H. Lurie Comprehensive Cancer Center, Chicago, IL 60611, USA

[4]Department of Biomedical Engineering, McCormick School of Engineering, Evanston, IL 60208, USA

Supplementary Figures

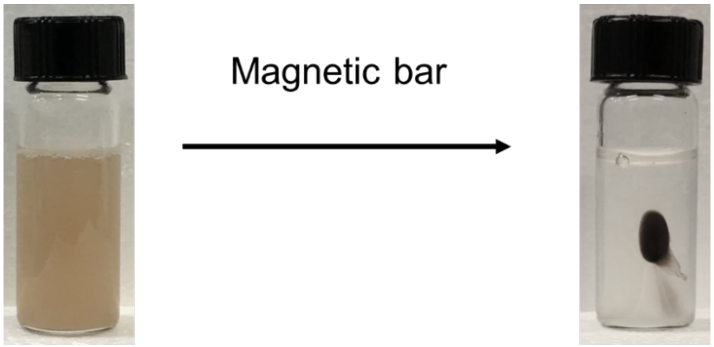

Supplementary Figure 1. Digital photos of hybrid core-shell vesicles collected by static magnetic field using a permanent magnet after being attached to the bottle for 2 h.

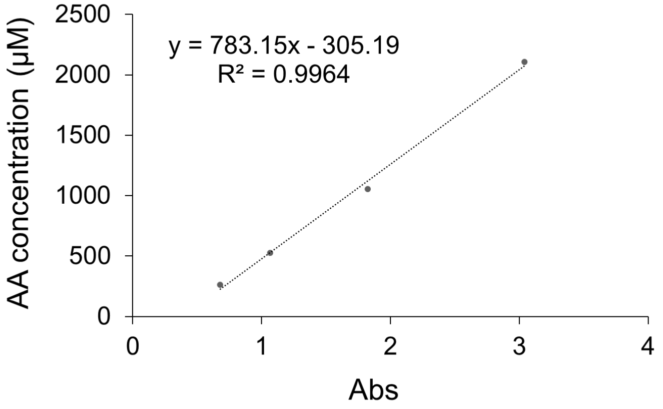

Supplementary Figure 2. UV standard curve of AA with various concentrations (at 260 nm). Source data are provided as a Source data file.

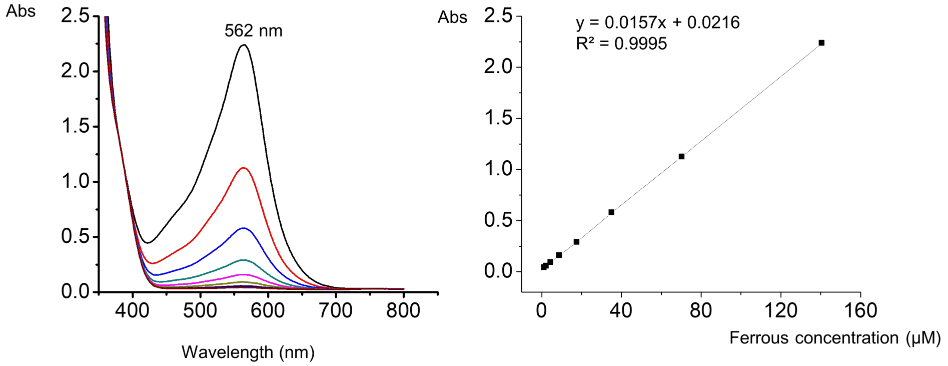

Supplementary Figure 3. UV-spectrum and standard curve of the mixture and various concentrations of ferrous state. Source data are provided as a Source data file.

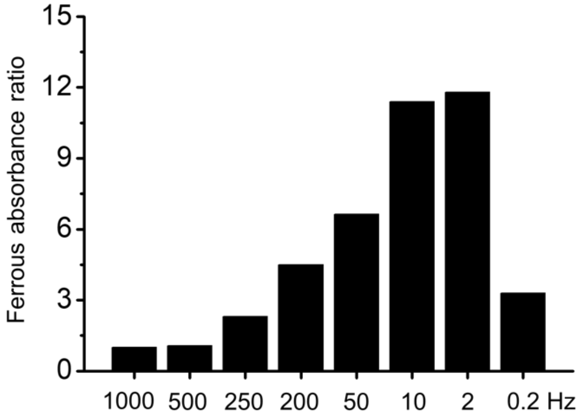

Supplementary Figure 4. The relative concentration change of Ferrous ions tested by ferrous probe at 4 h post treatment of MF in various frequency for 10 min. 2 Hz enabled the highest generation of ferrous. Source data are provided as a Source data file.

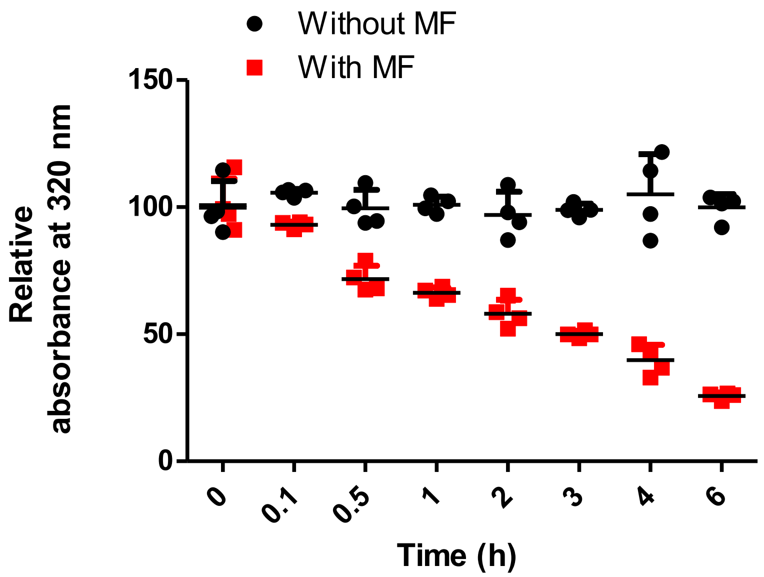

Supplementary Figure 5. Relative absorbance change of the HCSVs treated with or without magnetic fields (2 Hz) at 320 nm. Although soluble ferric ions have strong absorbance and provide sufficient spectra to analyze its concentration, calculating the ferric ions in the mixture of ions and HCSVs is still difficult because of the absorbance of IONCs on HCSVs. In addition, the absorbance of soluble ferrous ions is weak and not useful at the levels of ferrous iron typically present in groundwater. In contrast, ferric ions show a broad absorbance peak arranged from 300 nm to 400 nm.[1] Thus, the change of relative absorbance at 320 nm could partially be used to measure the reduction of ferric ions in the mixture. n=4 independent samples. Data are shown as means ± s.d. Source data are provided as a Source data file.

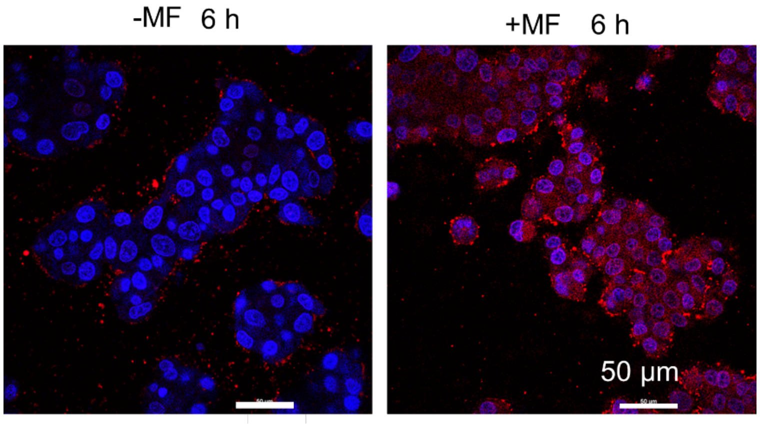

Supplementary Figure 6. Confocal images of cancer cells treated with doxorubicin hydrochloride (Dox) loaded hybrid core-shell vesicles, then follow-up treatment of MF. DAPI: Blue color, Red: Dox. Experiments were repeated three times.

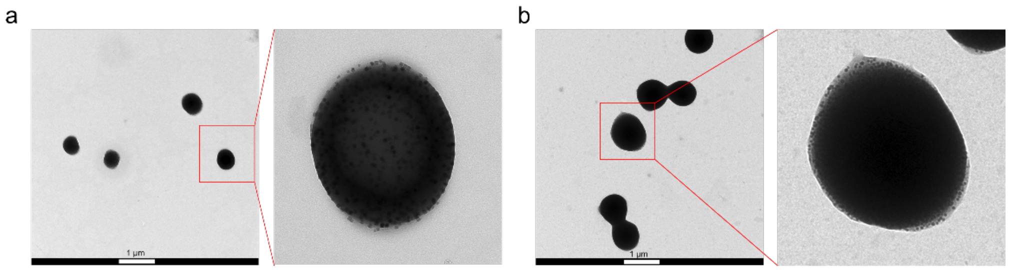

Supplementary Figure 7. TEM images of hybrid core-shell vesicles (a) before the MF treatment (scale bar: 1 µm). and (b) after the MF treatment. Source data are provided as a Source data file. Experiments were performed one time.

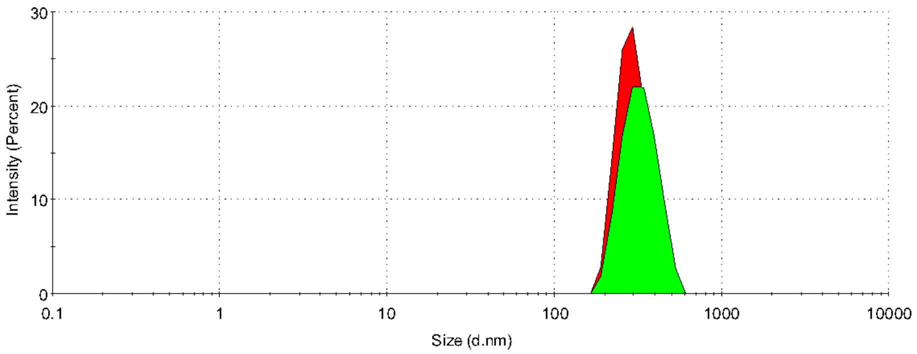

Supplementary Figure 8. DLS analysis of hybrid core-shell vesicles with thin shell (red) and hybrid core-shell vesicles with thick shell (green). There is a single peak with the size range 300 nm.

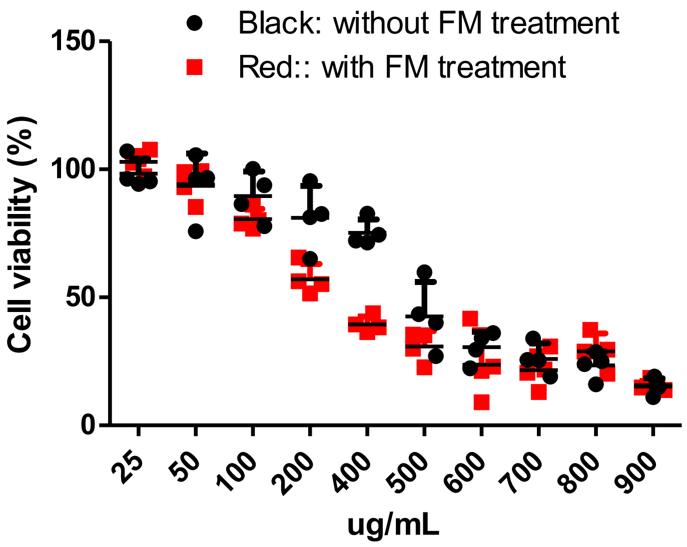

Supplementary Figure 9. TRAMP-C1 cells treated with various concentrations of HCSVs with/without MF. n=4 independent samples. Data are shown as means ± s.d. Source data are provided as a Source data file.

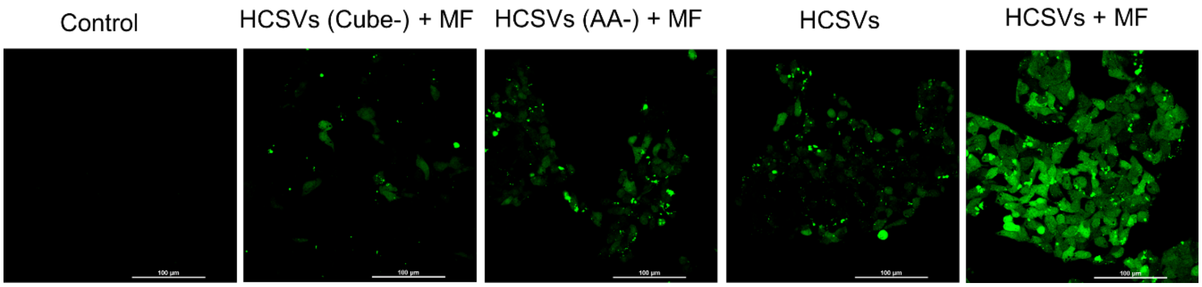

Supplementary Figure 10. ROS accumulation in TRAMP-C1 cells after various treatments. More brighter of green color means more higher level of ROS. Source data are provided as a Source data file. (scale bar: 100 μm). Experiments were repeated three times.

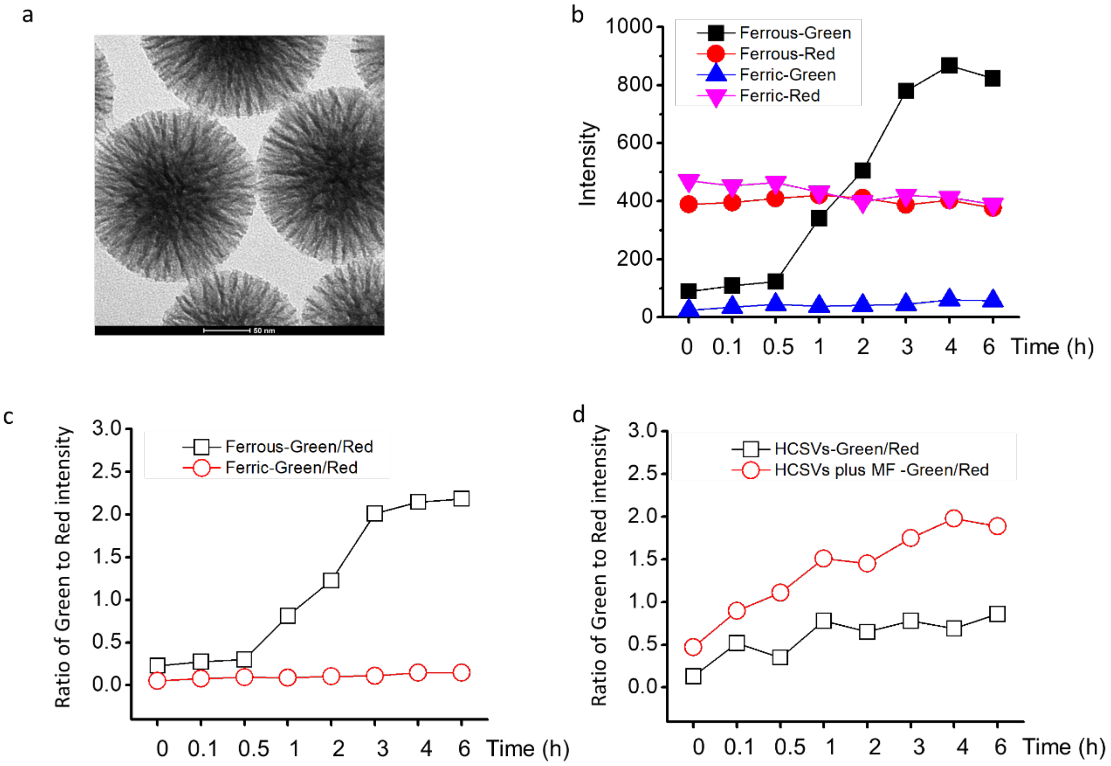

Supplementary Figure 11. Lipid peroxide stained with fluorescent C11-BODIPY581/591 in cells after coincubation with ferrous/ferric ions loaded mesoporous silica nanoparticles (MSN). (a) TEM images of MSN. Experiments were repeated three times. (scale bar: 50 nm). (b) Fluorescence emission intensity of C11-BODIPY581/591 at 530 nm (green) and 591 nm (red) in cells after coincubated with ferrous-MSN (Ferrous-Green or Ferrous-Red) or ferric-MSN (Ferric-Green or Ferric-Red). (c) Ratio of green:red fluorescence intensity in cells after coincubated with ferrous-MSN or ferric-MSN. (d) Ratio of green:red fluorescence intensity in cells after coincubated with HCSVs following with/without MF treatment. Source data are provided as a Source data file.

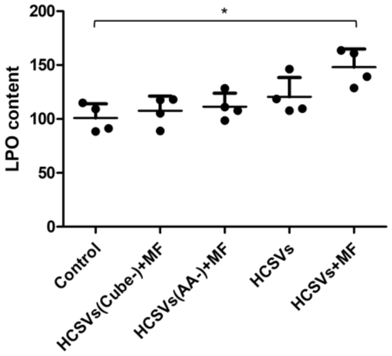

Supplementary Figure 12. LPO content of TRAMP-C1 cells treated with PBS, HCSVs (Cubes-) plus MF (2 Hz 10 min), HCSVs (AA-) plus MF, HCSVs, HCSVs plus MF. n=4 independent samples. *p=0.0176. Data are shown as means ± s.d. $p < 0.05$, two-tailed paired t-test. Source data are provided as a Source data file.

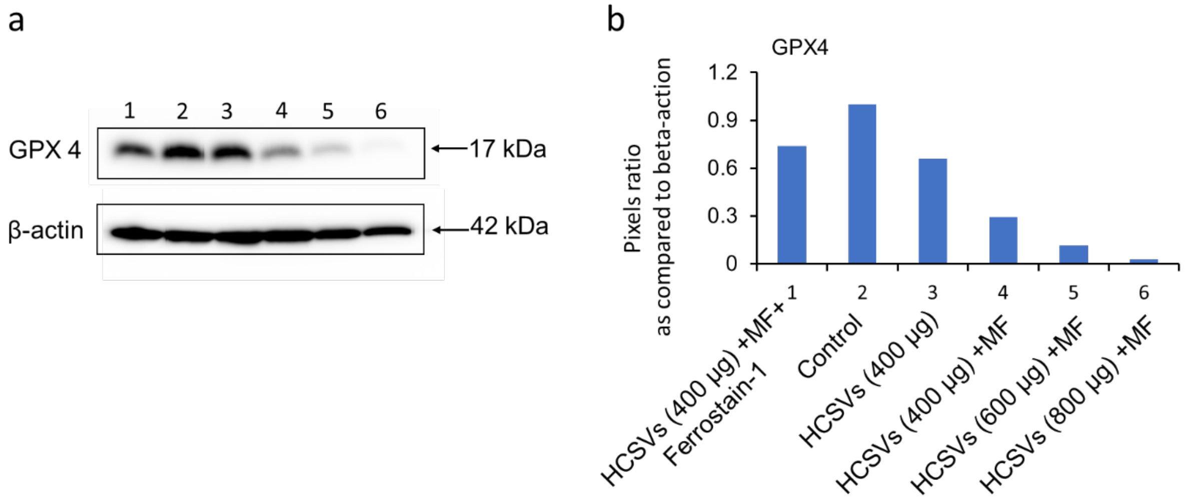

Supplementary Figure 13. (a) Western blot analysis of Tramp-C1 cells treated with various methods for 12 h. Experiments were performed one time. (b) Quantification of relative bands of western blotting. Lane 1 to Lane 6 were treated with HCSV(400 μg)+MF+ Ferrostain-1, Control, HCSVs (400 μg), HCSVs (400 μg)+MF, HCSVs (600 μg)+MF, HCSVs (800 μg)+MF, respectively. HCSVs (400 μg): 400 μg/mL of HCSVs, HCSVs (600 μg): 600 μg/mL of HCSVs, HCSVs (800 μg): 800 μg/mL of HCSVs. Quantitative comparisons were performed between samples on the same gels/blots. Source data are provided as a Source data file.

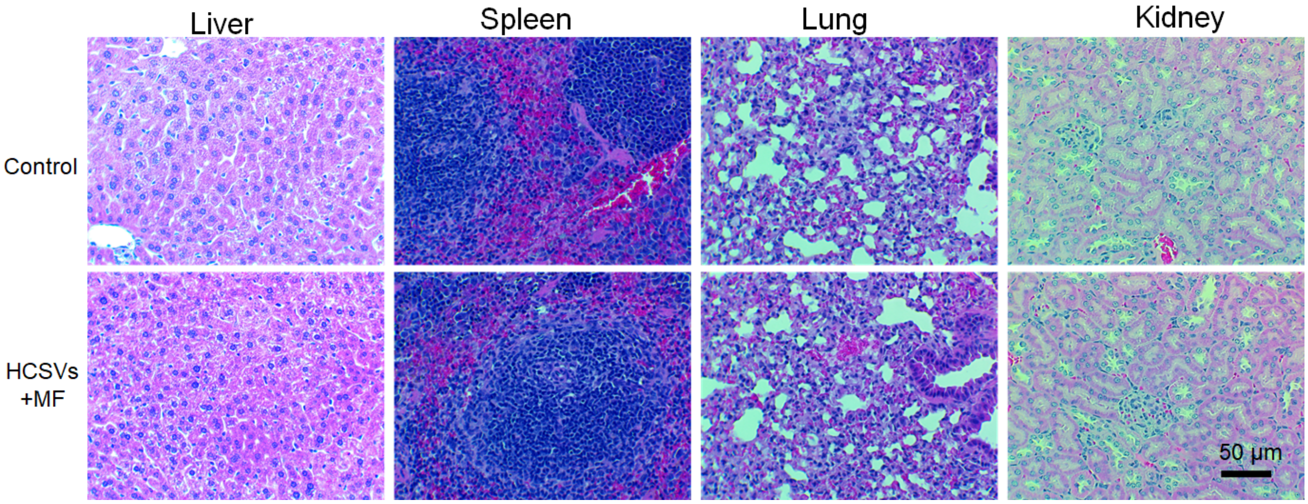

Supplementary Figure 14. H&E-stained organ (liver, spleen, lung, and kidneys) slices collected 14 days after various treatment (control: mice without treatment, HCSVs+MF: mice treated with combination of MF and HCSVs as described *in vivo* treatment). The results demonstrated that there was no noticeable tissue damage in the collected organs. Experiments were performed one time.

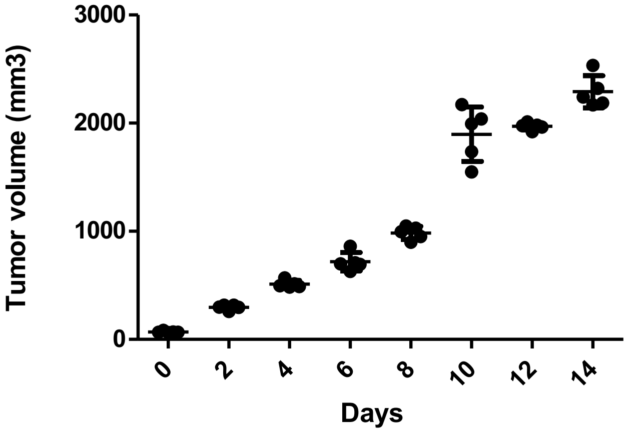

Supplementary Figure 15. Tumor growth curves of the TRAMP-C1 bearing mice treated with intravenous injection (I.V.) of HCSVs (50 μL, 10 mg/Kg) following with MF treatment for 14 days post I.V. (n=5 independent samples) Those mice were repeatedly treated three times at 0-day, 3-day, and 6-day time point. Data are shown as means ± s.d. Source data are provided as a Source data file.

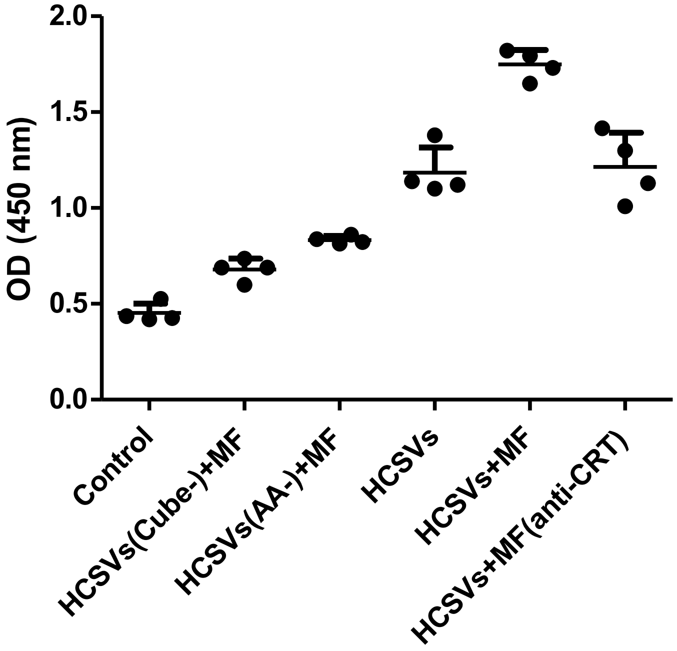

Supplementary Figure 16. Splenocytes proliferation assay. Splenocytes obtained from treated mice were re-stimulated by antigens, and the cell proliferation was measured by a CCK-8 kit assay. n=4 independent samples. Data are shown as means ± s.d. Source data are provided as a Source data file.

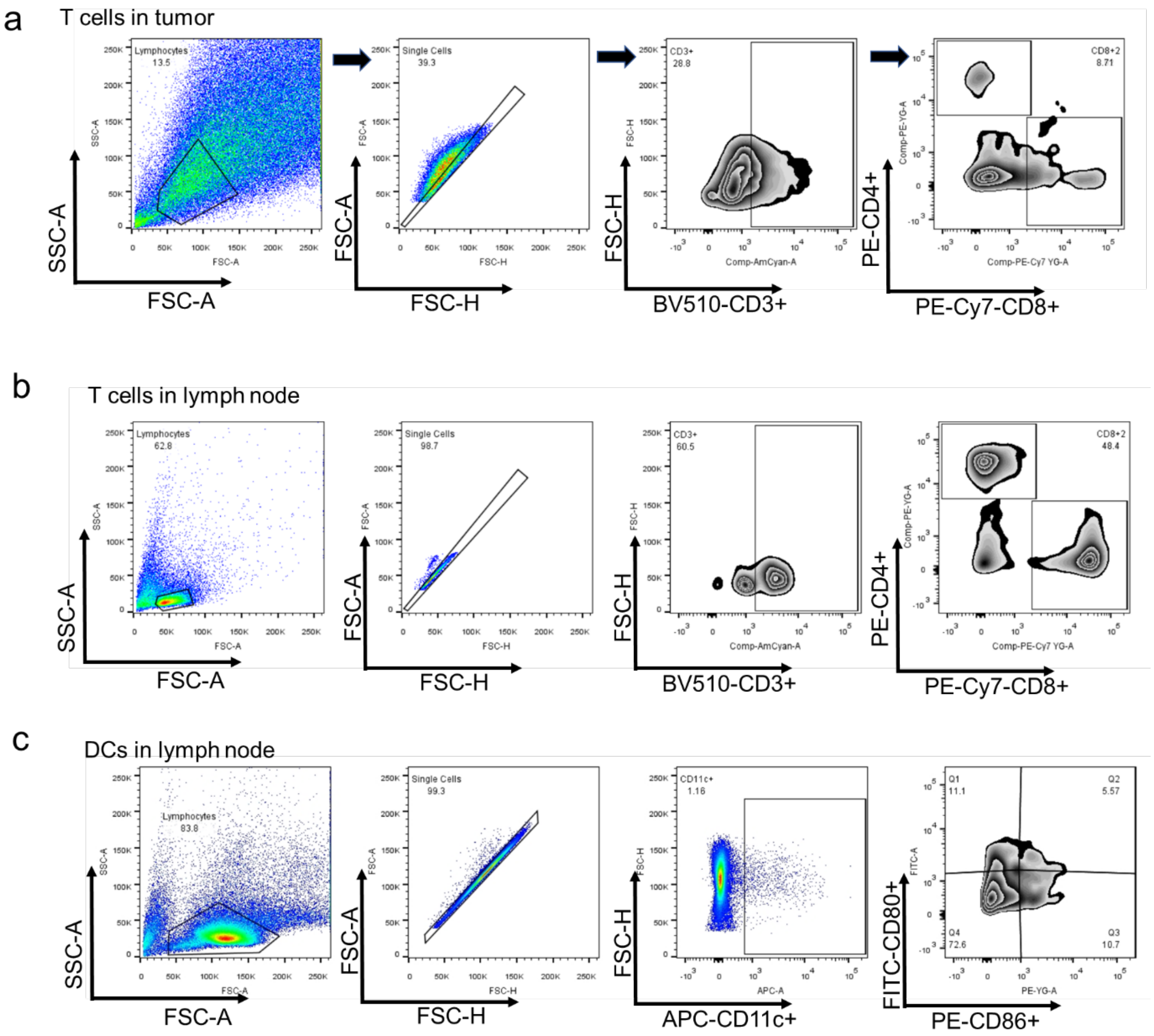

Supplementary Figure 17. Gating strategies used for cell sorting. (a) intra-tumoral infiltration of CD8+(CD3+CD4-CD8+), (b) CD8+ (CD3+CD4-CD8+) T cells in lymph node, (c) mature Dendritic cells in lymph node (CD11c+ CD80+CD86+, CD11c+ CD80, and CD11c+CD86+).

Reference:

1. Lohani CR, Lee KH. The effect of absorbance of Fe^{3+} on the detection of Fe^{3+} by fluorescent chemical sensors. *Sensor Actuat B-Chem* **143**, 649-654 (2010).
